# Comparative assessment of antibacterial drugs used at the hospital level before and during COVID-19, according to the WHO AWaRe classification

**DOI:** 10.3389/fphar.2025.1642830

**Published:** 2025-09-01

**Authors:** Aigerim A. Balapasheva, Gaziza A. Smagulova, Aigul Z. Mussina, Daniyar S. Dilmagambetov, Kalzhan Zh. Yermekbayeva, Mirsaid S. Kelimberdiev, Mariya S. Kulnazarova, Lazzat S. Balymbetova, Liliya E. Ziganshina

**Affiliations:** ^1^ Department of Pharmacology, Clinical Pharmacology, West Kazakhstan Marat Ospanov Medical University, Aktobe, Kazakhstan; ^2^ Department of Otorhinolaryngology, Ophthalmology, Phthisiology and Dermatovenereology Department, West Kazakhstan Marat Ospanov Medical University, Aktobe, Kazakhstan; ^3^ Deparment of Surgical Diseases No 1, West Kazakhstan Marat Ospanov Medical University, Aktobe, Kazakhstan; ^4^ Department of Hospital Epidemiology, Medical Parasitology and Tropical Diseases, Russian Medical Academy of Continuous Professional Education, Moscow, Russia; ^5^ Department of General and Clinical Pharmacology, Рeoples’ Friendship University of Russia named after Patrice Lumumba (RUDN University named after Patrice Lumumba), Moscow, Russia; ^6^ Department of Pharmacology, Kazan State Medical University, Kazan, Russia

**Keywords:** antibacterial drugs, antibiotic consumption, AWaRe, world health organization, antibiotics, COVID-19

## Abstract

Antimicrobial resistance is a major global challenge, and the World Health Organization (WHO) promotes the Access, Watch, and Reserve (AWaRe) classification to support rational antibiotic use. In Kazakhstan, irrational prescribing remains common, and the COVID-19 pandemic intensified the misuse of broad-spectrum antibiotics. A retrospective study at a hospital in Aktobe (2019–2021) analyzed antibiotic use from pharmacy and medical records using the Anatomical Therapeutic Chemical/Defined Daily Dose (ATC/DDD) methodology, with categorization by the WHO AWaRe classification. The analysis revealed a persistent predominance of “Watch” antibiotics, particularly cephalosporins, fluoroquinolones, and carbapenems, with their use intensifying during the COVID-19 pandemic. The share of “Access” antibiotics remained well below the WHO-recommended target of 60%, fluctuating between 24% and 27% across the study period. Reserve antibiotics were not prescribed. The findings reveal gaps in antibiotic stewardship in Kazakhstan: overreliance on “Watch” antibiotics and underuse of “Access” drugs, contrary to WHO guidance. Implementing the AWaRe framework with national targets and monitoring is essential, and the study adds regional evidence to the global AMR discourse, underscoring the need for stronger policies, especially during pandemics.

## Introduction

Antibiotic resistance is a global public health concern with profound social and economic consequences. In many countries, pharmaceutical expenditures constitute 40%–70% of national health budgets (World Health Organization, 2017). Improving the rational use of antibiotics, particularly in hospital settings, is a key objective of the WHO Global Action Plan on Antimicrobial Resistance. The limited development of new antibiotics, coupled with the withdrawal of major pharmaceutical companies from the antimicrobial market, has prompted the WHO to issue warnings about the growing threat of supercritical pathogens and the urgent need to balance access to essential antibiotics with efforts to curb resistance (The State of the World’s Antibiotics, 2021).

In Kazakhstan, despite a recent slight decline in the overall consumption of systemic antibacterial agents, irrational use remains prevalent. Notably, 27.5% of antibiotics are taken without a doctor’s prescription, and antibiotics account for 29.9% of all prescriptions—significantly exceeding the WHO-recommended threshold of 20% (Zhussupova et al., 2020). The COVID-19 pandemic, which began in 2020, further exposed the problem of inappropriate antibiotic use, particularly in Kazakhstan. During this period, antibiotics were frequently prescribed without clinical justification, and the country lacked comprehensive systems for epidemiological surveillance and antimicrobial resistance monitoring in healthcare institutions (Lavrinenko et al., 2023). International studies have similarly shown that while 72% of hospitalized COVID-19 patients received antibiotics, only 8% had confirmed bacterial or fungal coinfections (Haileyesus et al., 2020; Toner et al., 2015; Prestinaci et al., 2015). Experts warn that we are approaching an era in which many antibiotics may become ineffective due to widespread resistance (Tacconelli et al., 2018).

To address these challenges, the WHO developed the AWaRe classification—categorizing antibiotics into Access, Watch, and Reserve groups. This framework serves as a global stewardship tool for improving antibiotic selection, expanding access to essential medicines, and reducing resistance. The WHO has set a target: by 2023, at least 60% of national antibiotic consumption should come from the “Access” category to ensure safer and more effective treatment and mitigate the spread of resistance (World Health Organization, 2018). However, the COVID-19 pandemic has hindered progress toward this goal, with recent data indicating a growing prevalence of resistance to key antibiotics, including carbapenems and polymyxins (Gaspar et al., 2021; De Carvalho Hessel Dias et al., 2021).

Analyzing patterns of antibiotic consumption in healthcare institutions is essential for understanding the current situation and developing evidence-based strategies to combat resistance. The present study aims to conduct a comparative assessment of systemic antibiotic use in a hospital in Aktobe, Kazakhstan, from 2019 to 2021, using the WHO AWaRe classification. The primary goal is to justify the need for implementing the AWaRe classification system in Kazakhstan as a practical management tool for optimizing antibiotic use and establishing performance indicators.

## Materials and methods

This study employed a retrospective observational design, analyzing antibiotic consumption data from the hospital of Aktobe, Kazakhstan, over 3 years (2019–2021). Data were collected from the “1C: Accounting” program and the pharmacy database, focusing on the “Movement of Medicines in the Organization” section. All antibacterial agents were classified according to the WHO AWaRe classification, utilizing the 2021 version, which is updated biennially.

Data on antibiotic use from 2019 to 2021 were obtained from three complementary primary sources. First, dispensing records were extracted from the “1C: Accounting” pharmacy module, capturing all systemic antibacterial releases (dosage form, strength, and quantity). Second, electronic medical records provided admission and discharge dates for each patient, as well as detailed prescription information (daily dose, dosing frequency, and treatment duration). Third, to eliminate entry errors and duplicate counts, pharmacy dispensation data were cross-checked against the clinical pharmacist’s prescription logs.

All exported data were merged into a single dataset using each patient’s unique identifier and prescription date. Data integrity and completeness were overseen by the lead pharmacist - who verified consistency between dispensed and prescribed doses—and the clinical pharmacologist–who confirmed correct classification of dosage forms and dosages and validated the PDD and DDD calculations. This multi-step collection and validation process ensures reliable pharmacoepidemiological analysis in accordance with the ATC/DDD methodology and WHO AWaRe classification.

The ATC/DDD (Anatomical Therapeutic Chemical/Defined Daily Dose) methodology was used to conduct a pharmacoepidemiological study of consumed antibacterial drugs from 2019 to 2021. This methodology, recommended by the World Health Organization (WHO) as an international standard, is widely used to assess the use of medicines. WHO refers to it as the “gold standard” because it provides a unified approach to data analysis, allows for comparisons of drug consumption across different regions and countries, and tracks the dynamics of their use over time. The ATC/DDD method not only helps assess consumption volumes but also identifies trends and develops strategies for the rational use of medicines.

In accordance with the ATC/DDD methodology, the international nonproprietary names (INN) of all antibacterial drugs consumed from 2019 to 2021 were determined based on their trade names (TN), and the corresponding ATC codes (Anatomical Therapeutic Chemical Classification) were assigned according to the State Register of Medicines. The data were obtained from the official website of the National Center for Expertise of Medicines and Medical Devices of the Republic of Kazakhstan (NDDA.KZ) (NLE, 2025).

Prescribed Daily Dose (PDD) and Defined Daily Dose (DDD) Calculations To align with WHO recommendations and account for treatment duration, we introduced the concept of Prescribed Daily Dose (PDD)—the mean actual daily dose prescribed, derived directly from dispensing records and EMR orders. We then compared each PDD to the WHO Defined Daily Dose (DDD) for that antibiotic.

1. Determine PDD for each antibiotic
- Extract from “1C: Accounting” the total quantity of each antibiotic dispensed (in grams or units) and the total number of prescription days (from EMR).
- Calculation is performed using Equation 1:

$${PDD}_{i}=\frac{Total\;amount\;of\;drug\;i\;dispensed}{Total\;prescription-days\;for}$$ (1)

2. Calculate patient-days
- For each patient j, calculate using Equation 2:

$${Patient-days}_{j}=\left({discharge_{date}}_{j}-{admission\_date}_{j}\right)+1$$ (2)

- The total number of patients (NNN) per year is determined by Equation 3:

$$Total\;patient-days=\sum_{j=1}^{N}{Patient-days}_{j}$$ (3)

3. Compute DDD per 100 patient-days
- For each antibiotic i, it is determined by Equation 4:

$$\frac{{PDD}_{i}}{WHO\;{DDD}_{i}}$$ (4)

- Aggregate data for all antibiotics and normalize by patient days using Equation 5:

$$DDD/100\;patient-days=\frac{\sum_{i}\left(\frac{{PDD}_{i}}{WHO\;{DDD}_{i}}\right)}{Total\;patient-days}\times 100$$ (5)

**FIGURE 1:**
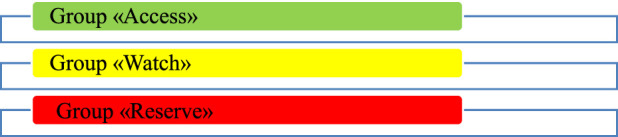
Classification of antibiotics by AWaRe groups.

Atraffic light color code shown in Figure 1 represents these categories:

4. Comparison with WHO targets
- Compare annual DDD/100 patient-days for each AWaRe group against the 60% (Access), 30% (Watch), and 10% (Reserve) benchmarks.

Example calculation for ceftriaxone (2020):

Total ceftriaxone dispensed: 38,086 g.

Total prescription-days: 2,000 days

$${PDD}_{ceftriaxone}=\frac{38086\;g}{2000\;days}=19.04\;g/day$$

WHO DDD(ceftriaxone): 2 g/day.

Total patient-days (2020):

11,682 patients×7 days≈81,774 patient−days

$$DDD\;per\;100\;patient-days=\frac{\left(\frac{19.04}{2}\right)}{81774}\times 100\approx 0.0116$$

After determining the ATC code for each of the antibacterial drugs used, the number of DDD (defined daily dose) per 100 hospital days was calculated. According to the WHO definition, DDD is the established average maintenance daily dose of a drug used for the main indication in an adult weighing 70 kg. For this purpose, the doses of all antibacterial products consumed in 2019–2021 (vials, tablets, ampoules, etc.) were determined, along with the total number of hospital days, DDD (average daily doses), and the ATC/DDD index in grams. The ATC/DDD index values in grams were obtained from the website of the WHO Collaborating Centre for Drug Statistics Methodology, which is updated every 2 years (WHO Collaborating Centre for Drug Statistics Methodology, 2025). This approach allowed for a comprehensive evaluation of antibiotic consumption patterns, facilitating the identification of trends in the use of “Access,” “Watch,” and “Reserve” antibiotics (Hoffman et al., 2015).

To assess the rationality of the use of antibacterial drugs consumed from 2019 to 2021, the AWaRe classification recommended by WHO, titled “AWaRe classification of antibiotics for evaluation and monitoring of use, 2021,” was utilized (World Health Organization, 2021).

The AWaRe classification, developed by the WHO, aims to reduce antibacterial resistance and enhance the safety and effectiveness of antibacterial use. By 2023, the WHO targets that at least 60% of antibacterial drugs prescribed in hospitals will be in the Access category, no more than 30% in the Watch category, and 10% in the Reserve category. A traffic light color code represents these categories:

### Access (green)

The Access group includes antibacterials that are effective against a broad spectrum of common pathogens with lower resistance potential. It comprises 48 antibiotics, 19 of which are listed in the WHO Model List of Essential Medicines as first- or second-choice options for treating specific infectious syndromes.

### Watch (yellow)

The Watch group consists of antibacterials with higher resistance potential, including critical drugs that are at risk of developing bacterial resistance. It contains 110 antibiotics, 11 of which are listed in the WHO Model List of Essential Medicines as first- or second-choice options for specific infectious syndromes.

### Reserve (red)

The Reserve group includes antibacterial drugs that are reserved for confirmed or suspected infections caused by multidrug-resistant organisms. These “last resort” options should be used only when all alternatives have failed. This group contains 22 antibiotics listed in the WHO Model List of Essential Medicines and should be prioritized in stewardship programs to maintain their effectiveness.

**FIGURE 2:**
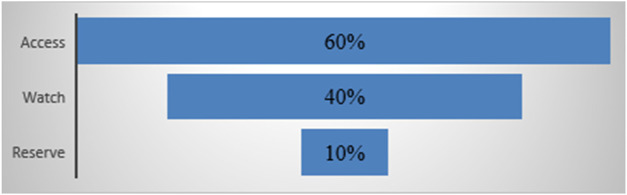
Strategic goals of WHO regarding the AWaRe classification.

The AWaRe classification promotes rational antibiotic use and improves access to essential medicines globally. It helps monitor antibiotic consumption, set targets, and track management policies to optimize use and reduce resistance. The WHO aims for Access antibiotics to account for at least 60% of national antibiotic consumption by 2023, as shown in Figure 2.

For the quantitative analysis of antibiotic consumption, the Defined Daily Dose (DDD) per 100 patient-days (DDD/100 patient-days) indicator recommended by the WHO was used to assess medication use in hospitals. The calculation of DDD for each antibiotic was performed in accordance with the ATC/DDD index established by the WHO at the time of analysis. The obtained DDD values were correlated with the total number of patient-days in the hospital for each year of the study (Table 1). This approach allowed for the evaluation of the consumption of each antibiotic and the overall consumption within each AWaRe group. The use of the DDD/100 patient-days indicator provides a more accurate analysis of consumption by taking into account actual bed occupancy.

**TABLE 1:** The indicator of treated patients in the hospital and the number of beds.

%No	Indicators	2019	2020	2021
1	Total number of treated patients in the hospital (adults only)	15,986	11,682	12,433
2	Of which: male	4,955	3,583	3,702
3	Of which: female	11,031	8,099	9,731
4	Number of beds (adults only)	400	400	400

Before the COVID-19 pandemic (2019), the Aktobe Medical Center was a multidisciplinary hospital with 400 beds for adult patients. Data were collected from 15 clinical departments covering 25 different medical specialties. During the pandemic (from April 2020), the hospital was repurposed as a provisional facility, maintaining 400 beds for adult patients (Order of the Aktobe Regional Health Department No. 68–5 dated 16 April 2020). A separate unit with 100 beds continued to operate for pregnant women, postpartum women, and mothers. The total number of patients receiving inpatient treatment in the provisional hospital in 2020–2021 was 2,223 individuals with severe pneumonia and comorbidities related to COVID-19 (Table 2). Only data on adult patients were included in the analysis.

**TABLE 2:** Consumption indicators of all medications and antibacterial agents from 2019 to 2021.

%No	Indicators	2019	2020	2021
1	Total medications	242	248	245
2	Of which: antibacterial agents	25	28	26

To determine the ATC code for systemic antibacterial agents, the code “J01”was used in accordance with the ATC classification. The category “J” denotes anti-infective agents for systemic use and includes medications used to treat infections caused by bacteria or other microorganisms. This category comprises the following subcategories: J01A- tetracyclines, J01B- phenicols, J01C - beta-lactam antibacterials (penicillins), J01D - other beta-lactam antibacterials, J01E - sulfonamides and trimethoprim, J01F - macrolides, lincosamides, and streptogramins, J01G - aminoglycoside antibacterials, J01M - quinolone antibacterials, J01R - combinations of antibacterial agents, and J01X - other antibacterial agents. Statistical processing of the obtained results was performed using Microsoft Excel software.

## Results

The Access category includes essential antibiotics that should be widely available for the treatment of common infections and used as first-line drugs. In 2019, antibiotics from the Access group accounted for 24.8% (95% CI 7.3%–40.7%) of total consumption (Table 3).

**TABLE 3:** List of J01 antibiotics consumed in 2019, according to the WHO AWaRe classification.

%No	ATC code	Antibiotic	Class	PDD (g/day)	WHO DDD (g/day)	AWaRe category	Included in the WHO list of essential medicines
1	J01GB06	Amikacin	Aminoglycosides	0.269	1.0	Access	yes
2	J01CA04	Amoxicillin sodium and potassium clavulanate	Penicillin group	0.363	1.5	Access	yes
3	J01FA10	Azithromycin	Macrolides	0.024	0.3	Watch	yes
4	J01CR02	Ampicillin sodium salt	Beta-lactam is a beta-lactamase inhibitor	0.363	2.0	Access	yes
5	J01CE01	Benzylpenicillin	Beta-lactam is a beta-lactamase inhibitor	0.164	3.6	Access	yes
6	J01XA01	Vancomycin	Glycopeptides	0.210	2.0	Watch	yes
7	J01GB03	Gentamicin	Aminoglycosides	0.003	0.24	Access	yes
8	J01DH04	Doripenem	Carbapenems	0.043	1.5	Watch	no
9	J01FA09	Clarithromycin	Macrolides	0.021	0.5	Watch	yes
10	J01MA12	Levofloxacin	Fluoroquinolones	0.978	0.5	Watch	no
11	J01FF02	Lincomycin	Lincosamides	0.278	1.5	Watch	no
12	J01DH02	Meropenem	Carbapenems	0.400	2.0	Watch	yes
13	J01XD01	Metronidazole	Nitroimidazoles	5.414	1.5	Access	yes
14	J01MA14	Moxifloxacin	Fluoroquinolones	0.003	0.4	Watch	no
15	J01MA01	Ofloxacin	Fluoroquinolones	0.148	0.4	Watch	no
16	J01DH51	Prepenem	Carbapenems	0.100	2.0	Watch	no
17	J01DD01	Cefotaxime	Cephalosporins (3rd generation)	6.001	2.0	Watch	yes
18	J01DD04	Ceftriaxone	Cephalosporins (3rd generation)	5.568	2.0	Watch	yes
19	J01DE01	Cefepime	Cephalosporins (4th generation)	0.499	2.0	Watch	no
20	J01DB04	Cefazolin	Cephalosporins (1st generation)	0.147	3.0	Watch	yes
21	J01MA02	Ciprofloxacin	Fluoroquinolones	1.789	1.0	Watch	yes
22	J01DC02	Cefuroxime	Cephalosporins (2nd generation)	0.688	1.5	Watch	yes
23	J01DD02	Ceftazidime	Cephalosporins (3rd generation)	0.459	2.0	Watch	yes
24	J01DH03	Ertapenem	Carbapenems	0.364	1.0	Watch	no
25	J01FA01	Erythromycin	macrolides	0.178	1.0	Watch	no

The Watch category includes antibiotics that have a higher potential for resistance and should be used more cautiously. Watch group antibiotics accounted for 75.2% (95% CI 59.3%–92.7%) of total consumption in 2019 and included 19 different agents (Table 3).

In 2020, the number of drugs in the Access category increased to eight. In addition to the drugs already in use, amoxicillin and thiamphenicol glycinate acetylcysteine were added. The share of drugs in this category rose to 27.6% (95% CI 11.3%–43.9%), calculated as a proportion of total DDDs. Confidence intervals were derived using the binomial proportion method. Another drug, piperacillin, was added to the Watch category, bringing the total number of drugs in this group to 20: azithromycin, vancomycin, doripenem, clarithromycin, levofloxacin, lincomycin, meropenem, moxifloxacin, ofloxacin, prepenem, cefotaxime, ceftriaxone, cefepime, cefazolin, ciprofloxacin, cefuroxime, ceftazidime, ertapenem, erythromycin, and piperacillin. The share of drugs in the Watch category in 2020 was 72.4% (95% CI 56.1%–88.7%) (Table 4).

**TABLE 4:** List of J01 antibiotics consumed in 2020, according to the WHO AWaRe classification.

No	ATC code	Antibiotic	Class	PDD (g/day)	WHO DDD (g/day	AWaRe category	Included in the WHO list of essential medicines
1	J01GB06	Amikacin	Aminoglycosides	0.378	1.0	Access	yes
2	J01CR02	Amoxicillin sodium and potassium clavulanate	Penicillins + β-lactamase inhibitor	2.026	1.5	Access	yes
3	J01FA10	Azithromycin	Macrolides	3.476	0.3	Watch	yes
4	J01CR02	Ampicillin sodium salt	Beta-lactam is a beta-lactamase inhibitor	0.181	2.0	Access	yes
5	J01CE01	Benzylpenicillin	Beta-lactam is a beta-lactamase inhibitor	0.175	3.6	Access	yes
6	J01XA01	Vancomycin	Glycopeptides	0.077	2.0	Watch	yes
7	J01GB03	Gentamicin	Aminoglycosides	1.811	0.24	Access	yes
8	J01DH04	Doripenem	Carbapenems	0.079	1.5	Watch	no
9	J01FA09	Clarithromycin	Macrolides	0.343	0.5	Watch	yes
10	J01MA12	Levofloxacin	Fluoroquinolones	3.389	0.5	Watch	no
11	J01FF02	Lincomycin	Lincosamides	0.302	1.5	Watch	no
12	J01DH02	Meropenem	Carbapenems	0.404	2.0	Watch	yes
13	J01XD01	Metronidazole	Nitroimidazoles	6.906	1.5	Access	yes
14	J01MA14	Moxifloxacin	Fluoroquinolones	0.023	0.4	Watch	no
15	J01MA01	Ofloxacin	Fluoroquinolones	0.246	0.4	Watch	no
16	J01DH51	Prepenem	Carbapenems	0.138	2.0	Watch	no
17	J01DD01	Cefotaxime	Cephalosporins (3rd generation)	6.151	2.0	Watch	yes
18	J01DD04	Ceftriaxone	Cephalosporins (3rd generation)	19.043	2.0	Watch	yes
19	J01DE01	Cefepime	Cephalosporins (4th generation)	0.553	2.0	Watch	no
20	J01DB04	Cefazolin	Cephalosporins (1st generation)	4.204	3.0	Watch	yes
21	J01MA02	Ciprofloxacin	Fluoroquinolones	2.872	1.0	Watch	yes
22	J01DC02	Cefuroxime	Cephalosporins (2nd generation)	0.718	1.5	Watch	yes
23	J01DD02	Ceftazidime	Cephalosporins (3rd generation)	1.275	2.0	Watch	yes
24	J01DH03	Ertapenem	Carbapenems	0.980	1.0	Watch	no
25	J01FA01	Erythromycin	macrolides	0.979	1.0	Watch	no
26	J01CA04	Amoxicillin	Antibiotic of the penicillin group	0.018	1.0	Access	yes
27	J01CR05	Piperacillin	Antibiotic, Penicillins + β-lactamase inhibitor	0.165	3.0	Watch	yes
28	J01B A02	Thiamphenicol glycinate acetylcysteinate	Amphenicols	0.005	2.0	Access	yes

These drugs accounted for 29.6% (95% CI 13.5%–45.7%) of total antibiotic consumption, based on DDD volume. Confidence intervals reflect the proportion of total DDDs consumed and were calculated using the binomial method. Confidence intervals were calculated using the binomial proportion method. The Watch category also included 20 drugs: azithromycin, vancomycin, doripenem, clarithromycin, levofloxacin, lincomycin, meropenem, moxifloxacin, ofloxacin, prepenem, cefotaxime, ceftriaxone, cefepime, cefazolin, ciprofloxacin, cefuroxime, ceftazidime, ertapenem, erythromycin, and piperacillin. The proportion of drugs in the Watch category in 2021 was 70.4% (95% CI 54.3%–86.5%), highlighting the need for cautious use of these antibiotics (Table 5).

**TABLE 5:** List of J01 antibiotics consumed in 2021, according to the WHO AWaRe classification.

No	ATC code	Antibiotic	Class	PDD (g/day)	WHO DDD (g/day)	AWaRe category	Included in the WHO list of essential medicines
1	J01GB06	Amikacin	Aminoglycosides	0.179	1.0	Access	yes
2	J01FA10	Azithromycin	Macrolides	3.456	0.3	Watch	yes
3	J01CE01	Benzylpenicillin	Beta-lactam is a beta-lactamase inhibitor	1.200	3.6	Access	yes
4	J01XA01	Vancomycin	Glycopeptides	0.456	2.0	Watch	yes
5	J01GB03	Gentamicin	Aminoglycosides	1.467	0.24	Access	yes
6	J01DH04	Doripenem	Carbapenems	0.878	1.5	Watch	no
7	J01FA09	Clarithromycin	Macrolides	0.677	0.5	Watch	yes
8	J01MA12	Levofloxacin	Fluoroquinolones	1.456	0.5	Watch	no
9	J01FF02	Lincomycin	Lincosamides	0.266	1.5	Watch	no
10	J01DH02	Meropenem	Carbapenems	0.689	2.0	Watch	yes
11	J01XD01	Metronidazole	Nitroimidazoles	4.579	1.5	Access	yes
12	J01MA14	Moxifloxacin	Fluoroquinolones	0.234	0.4	Watch	no
13	J01MA01	Ofloxacin	Fluoroquinolones	0.145	0.4	Watch	no
14	J01DH51	Prepenem	Carbapenems	0.432	2.0	Watch	no
15	J01DD01	Cefotaxime	Cephalosporins (3rd generation)	4.151	2.0	Watch	yes
16	J01DD04	Ceftriaxone	Cephalosporins (3rd generation)	10.129	2.0	Watch	yes
17	J01DE01	Cefepime	Cephalosporins (4th generation)	1.343	2.0	Watch	no
18	J01DB04	Cefazolin	Cephalosporins (1st generation)	3.200	3.0	Watch	yes
19	J01MA02	Ciprofloxacin	Fluoroquinolones	1.727	1.0	Watch	yes
20	J01DC02	Cefuroxime	Cephalosporins (2nd generation)	0.688	1.5	Watch	yes
21	J01DD02	Ceftazidime	Cephalosporins (3rd generation)	0.987	2.0	Watch	yes
22	J01DH03	Ertapenem	Carbapenems	1.098	1.0	Watch	no
23	J01FA01	Erythromycin	macrolides	0.878	1.0	Watch	no
24	J01CA04	Amoxicillin	Antibiotic of the penicillin group	1.189	1.0	Access	yes
25	J01CR05	Piperacillin	Antibiotic, Penicillins + β-lactamase inhibitor	0.989	3.0	Watch	yes
26	J01B A02	Thiamphenicol glycinate acetylcysteinate	Amphenicols	0.231	2.0	Access	yes

This absence of Reserve category antibiotic use is notable and should be interpreted within the framework of antimicrobial stewardship. Reserve antibiotics are designated by WHO for treating multidrug-resistant infections and should be used only when absolutely necessary. Their non-use in this hospital may reflect rational prescribing, effective infection control, or limited access. While this can be seen as a positive stewardship outcome, it also underlines the importance of maintaining readiness and access to these critical drugs when clinically indicated.

Recalculated antibiotic consumption indicators

Using the refined methodology with “patient-days” as the denominator, we obtained the following DDD/100 patient-days values:

2019: 18.42 DDD/100 patient-days
2020: 39.78 DDD/100 patient-days
2021: 35.24 DDD/100 patient-days

These figures represent the ratio given in Equation 6:

$$DDD/100\;patient-days=\frac{\sum_{i}\left(\frac{{PDD}_{i}}{WHO\;{DDD}_{i}}\right)}{Total\;patient-days}\times 100$$ (6)

where the total number of patient days for each year was calculated as shown in Equation 7:

$$\sum_{j}^{\;}\left({discharge}_{j}-{admission}_{j}+1\right)$$ (7)

Example calculation for ceftriaxone (2020):
Total ceftriaxone dispensed: 38,086 g.
Total prescription-days for ceftriaxone: 2,000 days

${\text{PDD}}_{\text{ceftriaxone}}=38\;086\;g\;/\;2\;000\text{ days}=19.043\;g/\text{day}$

WHO DDD_ceftriaxone_ = 2 g/day.
Total patient-days (2020) = 81,774 days

$${DDD/100\;patient-days}_{\text{ceftriaxone}}=\frac{19.043/2}{81774}\times 100\approx 100$$

Cephalosporins and fluoroquinolones were among the most used antibiotic classes in 2021. Notably, third-generation cephalosporins such as ceftriaxone maintained leading positions, which raises concerns about selective pressure and potential resistance (see Table 5).

Furthermore, there was an increase in the consumption of azithromycin, which amounted to 3.456 DDD/100 bed-days in 2021, confirming its importance in the treatment of bacterial infections, especially in the context of a pandemic. The consumption of levofloxacin also increased, reaching 1.456 DDD/100 bed-days, likely due to its use in treating complicated respiratory tract infections.

Thus, 2021 continued the trend of increasing antibiotic consumption, underscoring the need to monitor and manage the use of antibacterial drugs to prevent antibiotic resistance.

**FIGURE 3:**
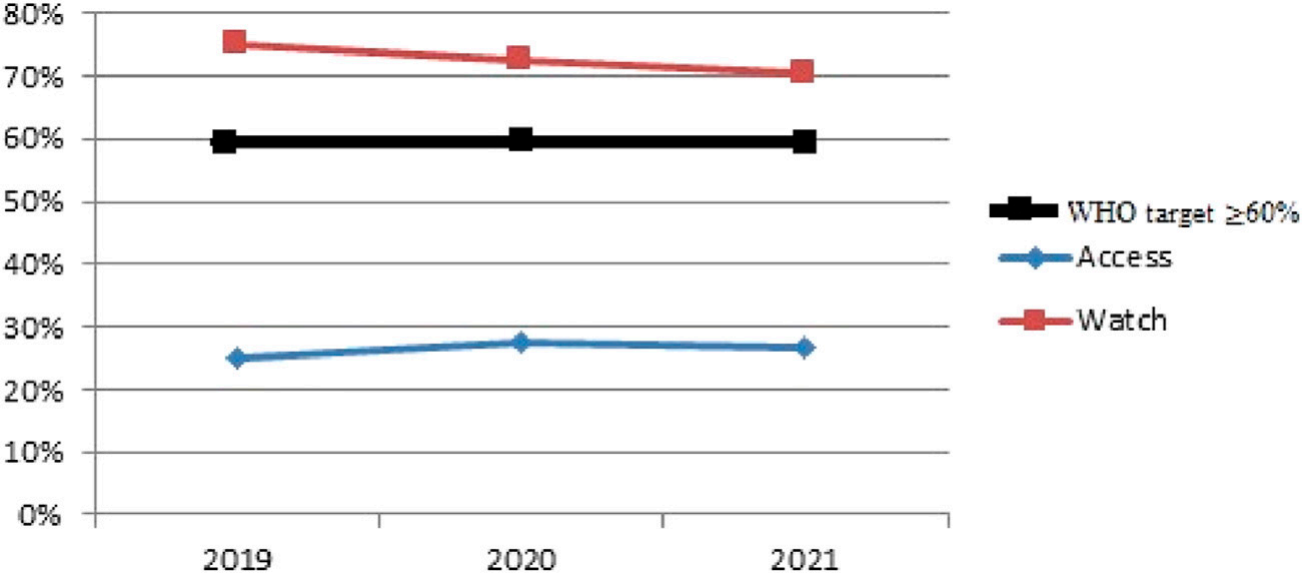
Comparative result of antibiotic consumption in Aktobe dispensary hospital, according to the AWaRe classification for 2019–2021. The horizontal line at 60% indicates the WHO-recommended target share for Access antibiotics.

**FIGURE 4:**
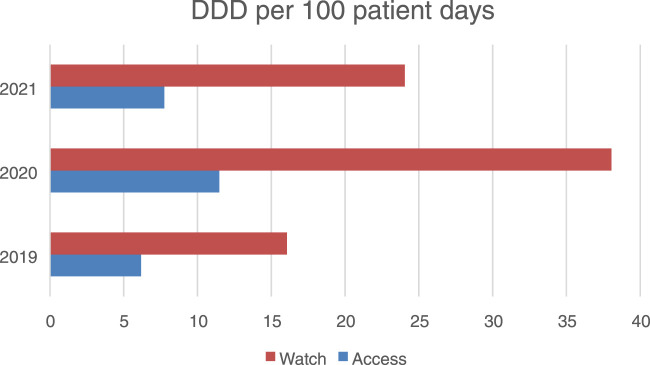
Antibiotic consumption in the access and watch groups in an hospital in Aktobe, Kazakhstan in 2019–2021 (in DDD per 100 bed days).

Based on the comparative analysis of systemic antibiotic consumption, the share of Access group antibiotics was calculated using total PDDs per year: in 2019–7.448 out of 23.574 PDDs (31.6%), in 2020–13.375 out of 55.198 PDDs (24.2%), and in 2021–8.445 out of 47.151 PDDs (17.9%). These figures indicate a downward trend in Access antibiotics over the 3-year period. Meanwhile, Watch antibiotics remained predominant (68.4%, 75.8%, and 82.1% respectively). This distribution forms the basis for Figures 3, 4 and served as input for the Cochran-Armitage trend test.

Using a chi-squared test, the proportions of antibiotics in the “Access” and “Watch” categories were compared across different years.

Chi-square test results:

Chi-squared value: 5.77

p-value: 0.056.

Degrees of freedom: two.

Expected frequencies:

Access: [30.38, 30.38, 28.25].

Watch: [69.62, 69.62, 64.75].

The chi-square value of 5.77 with a p-value of 0.056 suggests a possible trend in changes in antibiotic consumption over the years; however, this result does not reach the conventional threshold for statistical significance (p < 0.05). Therefore, while the data may indicate a potential shift, we cannot confidently reject the null hypothesis of stable consumption across the 3-year period. It is also important to note that in all 3 years, the proportion of Access antibiotics remained well below the WHO-recommended threshold of 60%, as shown by the reference line in Figure 3. This underperformance highlights a stewardship gap and underscores the need for improved antibiotic prescribing practices aligned with global guidelines.

In this regard, the observed variations in the proportions of Access and Watch category antibiotics from 2019 to 2021 should be interpreted with caution. The overall pattern appears relatively stable, and further data would be needed to confirm the presence of a statistically meaningful trend.

Despite fluctuations in antibiotic consumption, it can be concluded that there was a tendency towards more balanced use of antibiotics during the study period. This is a positive sign in the fight against antibiotic resistance.

To further analyze changes in antibiotic consumption in the Access category from 2019 to 2021, a Cochran-Armitage trend test was conducted.

The null hypothesis (H0) for the Cochran-Armitage trend test in this context is formulated as follows: There is no statistically significant trend in antibiotic consumption in the Access category between 2019 and 2021. This implies that any changes in antibiotic consumption in this category are not significant and can be attributed to random variation rather than actual changes in antibiotic prescribing practices.

1. Total number of DDDs (Equation 8):

$$N=n_{1}+n_{2}+n_{3}=6.576+11.500+8.845=27.921$$ (8)

where, N–total number of DDD;

$n_{1}$ – number of DDDs in 2019;

$n_{2}$ – number of DDDs in 2020;

$n_{3}$ – number of DDDs in 2021;

2. Average value (Equation 9):

$$\bar{X}=\frac{N}{3}=\frac{27.921}{3}=9.307$$ (9)

where, $\bar{X}$ – average value.

3. Standard deviation (Equation 10):

$$S=\sqrt{\frac{{\left(n_{1}-\bar{X}\right)}^{2}+{\left(n_{2}-\bar{X}\right)}^{2}+{\left(n_{3}-\bar{X}\right)}^{2}}{3-1}}=\sqrt{\frac{7.448+4.81+0.213}{2}}=2.494$$ (10)

where, S – standard deviation.

4. Calculate Z for each value (Equation 11):

$$Z_{n}=\frac{n_{n}-\bar{X}}{S}$$ (11)

$$Z_{1}=-1.095;\;Z_{2}=0.883;\;Z_{3}=-0.185;$$

where, Z – value for test.

5. Вычисление Q (Equation 12):

$$Q=\frac{{\left(Z_{1}+Z_{2}+Z_{3}\right)}^{2}}{3}=\frac{0.1576}{3}=0.0525$$ (12)

where, Q – value calculated to test the null hypothesis.

For a significance level of 0.05 and 1 degree of freedom, the critical value of chi-square is 3.841. Since Q ≈ 0.0525 < 3.841, we cannot reject the null hypothesis.

Thus, the analysis of antibiotic consumption in the Aktobe hospital for the period 2019–2021 revealed important trends in the use of drugs of the Access and Watch categories according to the AWaRe system. Despite fluctuations in consumption, there is a desire for a more balanced use, which can reduce the risk of antibiotic resistance. The increase in the share of Access antibiotics in 2020 and a slight decrease in 2021, along with the high level of use of Watch drugs, highlight the need for continuous monitoring and optimization of antibiotic therapy to effectively treat infections and maintain antibiotic efficacy.

The obtained results are limited to the analysis of data for 2019–2021, but available publications for subsequent years confirm the continuation of the general trend towards the predominance of Watch group antibiotics in clinical practice. Thus, according to the study by Kuzdenbaeva R.S. et al., in 2022, a number of hospitals in Kazakhstan also noted a high level of consumption of cephalosporins and fluoroquinolones, accounting for more than 70% of the total volume of antibiotics (Kuzdenbaeva et al., 2020). In addition, the review by Lavrinenko et al., 2023 emphasizes that even in the context of stabilization of the epidemiological situation, irrational prescription of antibiotics in COVID and post-COVID practice, including reserve drugs, remains (Lavrinenko et al., 2023). These data confirm the need to continue monitoring beyond the pandemic period and strengthen antimicrobial surveillance.

## Discussion

The issue of global antimicrobial resistance (AMR) and its impact on mortality and the economy is underscored by numerous international and national initiatives (World Health Organization, 2017; The State of the World’s Antibiotics, 2021). In Kazakhstan, where anti-infective agents represent the most frequently utilized class of pharmaceuticals (Zhussupova et al., 2020), the problem of irrational antibiotic use is particularly pressing. Our study, conducted at the Aktobe hospital from 2019 to 2021, corroborates this concern by revealing negative trends in antibiotic consumption, necessitating immediate action.

The results showed a fluctuation in the proportion of antibiotics classified as “Access,” increasing from 24% in 2019 to 27.6% in 2020, followed by a slight decrease to 26.9% in 2021. This trend is concerning as it is contrary to the World Health Organization target of 60% (World Health Organization, 2018). These rates of “Access” antibiotics pose a significant risk of exacerbating the problem of antibiotic resistance, which is already a pressing issue worldwide (World Health Organization, 2019). At the same time, the consumption of “Watch” antibiotics remains high. In 2019, the use of “Watch” antibiotics was 76%, decreasing to 72.4% in 2020, and then increasing again to 73.1% in 2021. The most commonly used antibiotics were cephalosporins, fluoroquinolones, and carbapenems. This observed trend is confirmed by a parallel study (Ablakimova et al., 2023) examining bacterial isolates from pneumonia patients in Aktobe, which revealed significant resistance to fluoroquinolones and cephalosporins, particularly among strains isolated from COVID-19 PCR + patients. This suggests that the increased consumption of “Watch” antibiotics may be a direct response to rising resistance among prevalent pathogens.

Notably, the chi-squared test yielded a p-value of 0.056, which is close to the threshold of statistical significance (p = 0.05). This suggests that the observed changes in antibiotic consumption patterns may be random and require further validation in larger studies. However, even in light of the lack of strict statistical significance, the observed trend of decreasing “Access” antibiotic use and increasing “Watch” antibiotic use raises significant concerns.

In 2020, due to the repurposing of the hospital as an infectious disease facility in response to COVID-19 (Order of the Aktobe Region Health Department No. 68–5 dated 16 April 2020), there was a notable surge in the consumption of “Watch” antibiotics, including ciprofloxacin, piperacillin/tazobactam, ertapenem, moxifloxacin, imipenem/cilastatin, and lincomycin, which had not been registered for use in 2019 and are not included in the WHO’s list of essential medicines.

The findings from our study highlight a critical gap in antibiotic stewardship practices, particularly in the context of the COVID-19 pandemic. Minor fluctuations in the use of Access and Watch antibiotics not only contravene WHO recommendations but also pose a significant risk of exacerbating antibiotic resistance, which is already a pressing problem worldwide. The increased reliance on Watch antibiotics in this study reflects a broader trend observed in different regions where the pandemic has changed prescribing habits and increased pressure on health systems.

Moreover, the implications of our findings extend beyond local practice, echoing global concerns about AMR and the urgent need for effective interventions. The observed patterns suggest that healthcare providers may be resorting to broader-spectrum antibiotics due to diagnostic uncertainties or a lack of rapid testing capabilities for bacterial infections in the context of viral pandemics. This underlines the necessity for improved diagnostic tools and clinical guidelines that can assist healthcare professionals in making informed decisions about antibiotic use.

Although the primary objective of this study was to assess the pattern of systemic antibiotic consumption according to the AWaRe classification, a more comprehensive analysis requires consideration of microbiological data reflecting the spectrum of pathogens and their resistance, as well as the severity of patient conditions and clinical outcomes.

In this context, the study by Ablakimova et al., in 2023, conducted at medical institutions in Aktobe, where our observation object was also located, is of particular importance. In this work, the microbial landscape and antibiotic resistance in patients with bacterial pneumonia, including COVID-19 positive ones, were assessed. It was found that the most common pathogens were *K. pneumoniae* (35.2% of all isolates) and *Acinetobacter* baumannii (28.3%). At the same time, *Klebsiella pneumoniae* demonstrated resistance to fluoroquinolones in 84% of cases and to third-generation cephalosporins in 76% of cases, and A. baumannii was resistant to carbapenems in 92% of cases (Ablakimova et al., 2023). These data suggest that the high proportion of watch antibiotic consumption, particularly cephalosporins and fluoroquinolones, reflects not only suboptimal empirical therapy regimens but also a possible response to resistant pathogens circulating in the hospital.

In addition, it is important to take into account the severity of the patient’s condition during the pandemic. According to our data, in 2020, 2,223 patients with severe pneumonia were hospitalized in the temporary hospital, most of whom had comorbidities (diabetes mellitus, obesity, cardiovascular pathologies). The average duration of hospitalization was 7 days, while about 28% of patients received combination antibacterial therapy (two or more active substances simultaneously). The frequency of carbapenem use (meropenem, doripenem, ertapenem) increased from 0.91 DDD/100 bed-days in 2019 to 2.2 DDD/100 bed-days in 2021, which may indicate more severe clinical forms and the need for escalation therapy.

Thus, the inclusion of microbiological data and characteristics of the severity of the patient’s condition allows for a deeper interpretation of the observed changes in antibiotic consumption. The high level of use of the Watch group drugs may be partly justified by clinical necessity in the context of limited availability of rapid bacterial diagnostics and the increasing number of severe cases of COVID-19. However, the lack of systematic monitoring of pathogen susceptibility and unified clinical algorithms contributes to irrational use of antibacterial drugs, which emphasizes the relevance of recommendations for the implementation of antimicrobial surveillance programs and the expansion of microbiological support for therapy.

In light of these findings, our study emphasizes the importance of implementing comprehensive antimicrobial stewardship programs that align with international best practices. The recommendations outlined earlier are crucial not only for improving antibiotic use in Kazakhstan but also for contributing to global efforts to combat AMR. By fostering a culture of responsible antibiotic prescribing and use, we can mitigate the risks associated with rising resistance levels and safeguard the effectiveness of existing antibiotics for future generations.

The results obtained are consistent with global trends observed in several studies. In light of these findings, it is clear that the trends observed in our study are part of a larger global challenge regarding AMR. Addressing these challenges requires a multifaceted approach, including improved diagnostic tools, clinical guidelines, and comprehensive antimicrobial stewardship programs.

Data presented in studies by other authors show that in Jordan, there was an 18% decrease in “Access” antibiotic consumption and a 26% increase in “Watch” antibiotic consumption in 2020 compared to 2019 (Al-Azzam et al., 2021). Similar trends have been noted in studies from other countries: a 37% increase in the use of “Watch” antibiotics in 2020 compared to 2019 in one study (Hussein et al., 2022), a 45.8% increase in the United Kingdom (Abdelsalam Elshenawy et al., 2023), and a statistically significant rise in the use of “Watch” and “Reserve” antibiotics in Turkey among hospitalized COVID-19 patients (Çalişkan et al., 2024). In Bangladesh, antibiotic sales increased by 31%, with a significant portion sold without prescriptions; notably, 54% of the ten most sold antibiotics fell into the “Watch” category (Orubu et al., 2021; Islam et al., 2022). A systematic review of antibiotic consumption across 76 countries from 2000 to 2015 indicated a 26.2% increase in “Access” antibiotics and a 90.9% increase in “Watch” antibiotics (Klein et al., 2021). In Kazakhstan, prior to the pandemic (2017–2019), there was also a notable use of “Watch” antibiotics, including reserve antibiotics such as linezolid (Zhussupova et al., 2021). A study conducted in Almaty revealed that most treatment methods during the COVID-19 pandemic did not align with national guidelines, particularly regarding antibiotic use (Kuzdenbaeva et al., 2020).

These findings highlight the urgent need for a cohesive strategy to address antibiotic stewardship in the context of rising antibiotic resistance levels. The results of this study highlight the urgent need for comprehensive measures to improve the rational use of antibiotics in Kazakhstan. These measures should include:

1. Implementation of the WHO AWaRe Classification Database. This will provide guidance for healthcare professionals on the rational prescribing of antibiotics.
2. Establishment of a National Monitoring and Surveillance System. A centralized system for monitoring antibiotic consumption and resistance patterns will enable the tracking of trends and the adjustment of strategies accordingly.
3. Strengthening Control Over Over-the-Counter Antibiotic Sales. It is essential to eliminate unrestricted access to antibiotics to minimize self-medication practices.
4. Increasing the Availability of Essential Antibiotics. Ensuring the registration and accessibility of “Access” category antibiotics, as well as pediatric and liquid formulations, is crucial.
5. Enhancing Healthcare Professionals’ Training. Mandatory training on the principles of rational antibiotic use and antimicrobial stewardship is a key factor in addressing this issue.
6. Public Awareness Campaigns. Extensive informational campaigns will help raise awareness about the AMR problem and the importance of rational antibiotic use.
7. Implementation of Antimicrobial Stewardship Programs in Hospitals. This will facilitate the monitoring of antibiotic prescriptions and adherence to evidence-based medicine.
8. Encouragement of Scientific Research. Supporting research on AMR, including the study of local resistance patterns, will aid in the development of new treatment strategies.
9. Improvement of Data Collection and Processing. Standardizing methods for collecting data on antibiotic use and resistance is necessary for informed decision-making.
10. International Collaboration. Partnering with international organizations, such as the WHO, will allow for the adaptation of best practices to the specific context of Kazakhstan.

The implementation of these recommendations will establish a robust system for managing antibiotic use, reduce antibiotic resistance levels, and enhance the resilience of Kazakhstan’s healthcare system. It is important to note that the necessity of these measures is supported by a meta-analysis conducted by Sulis et al. (Sulis et al., 2022), which demonstrated a correlation between antibiotic exposure and the emergence of multidrug-resistant bacteria, as well as a systematic review by Klein et al. (Zhussupova et al., 2021), highlighting a global trend of increasing use of “Watch” category antibiotics, particularly in low- and middle-income countries. Our study serves as further evidence of the urgency for adopting these measures in Kazakhstan.

## Ethical considerations

The ethical approval of the study was received from the local ethical commission of the NAO “West Kazakhstan Medical University named after Marat Ospanov”, Aktobe Kazakhstan [no.8, 10/15/2021] as part of a research project.

## Statistical analysis

Descriptive statistics in the form of absolute frequencies and proportions were used to analyze ATC/DDD, ABC/VEN, and AWaRe classification. Pearson chi-square and Z-tests were used to assess the distribution of categorical variables in groups before and during the COVID-19 pandemic. Given the categorical nature of the data, nonparametric tests were used in the analysis.

To analyze the DDD indicators, we used the Z-test to compare proportions, which is calculated by online calculators https://tiburon-research.ru/free-tools/z-test-calculator or a special Python programming language in Visual Studio. This test helps to check whether the differences between two proportions are statistically significant.

The test resulted in a Z-statistic and P-value, statistical analyses were considered significant when the P-value was below 0.05.

Data analysis was performed using IBM SPSS Statistics 22 software (SPSS Inc., Chicago, IL, United States), and GraphPad software (version 9.5.1, San Diego, CA, United States) was used to visualize the results.

## Conclusion

This study identifies concerning trends in antibiotic prescribing practices at the Aktobe dispensary hospital from 2019 to 2021. Despite a temporary increase in the use of “Access” group antibiotics in 2020, the overall proportion remained significantly below the WHO’s recommended 60% threshold. Instead, “Watch” antibiotics dominated the prescription patterns, accounting for 70.4%–76% of total antibiotic use, with high reliance on cephalosporins, fluoroquinolones, and carbapenems—agents associated with a greater risk of resistance development.

The COVID-19 pandemic further intensified the use of broad-spectrum antibiotics, including several not listed as essential by WHO. Although statistical analysis did not confirm a significant trend in consumption shifts, the persistent underuse of “Access” antibiotics and overuse of “Watch” group agents points to critical deficiencies in antimicrobial stewardship.

Addressing this issue requires a coordinated, multi-faceted strategy. Key recommendations include: implementing the WHO AWaRe classification database; establishing a national surveillance and monitoring system; enforcing regulations on over-the-counter antibiotic sales; ensuring access to essential antibiotics; enhancing professional training in antimicrobial stewardship; launching public awareness initiatives; and strengthening hospital-based antibiotic monitoring programs. Additionally, improving microbiological diagnostics and integrating local resistance data into clinical guidelines will support evidence-based prescribing.

These measures are vital for reducing antimicrobial resistance, improving treatment outcomes, and strengthening Kazakhstan’s healthcare system. Future research should expand the scope of analysis and include post-2021 data to track the long-term impact of stewardship interventions and pandemic-related prescribing shifts.

## Data Availability

The original contributions presented in the study are included in the article/supplementary material, further inquiries can be directed to the corresponding author.
